# Mitochondrial stress management: a dynamic journey

**DOI:** 10.15698/cst2018.10.158

**Published:** 2018-10-08

**Authors:** Miriam Valera-Alberni, Carles Canto

**Affiliations:** 1 Nestlé Institute of Health Sciences (NIHS), EPFL Innovation Park, 1015 Lausanne.; 2 School of Life Sciences, EPFL, 1015 Lausanne.

**Keywords:** mitochondria, mitochondrial dynamics, oxidative stress, unfolded protein response, mitophagy, mitochondrial biogenesis

## Abstract

Mitochondria undergo continuous challenges in the course of their life, from their generation to their degradation. These challenges include the management of reactive oxygen species, the proper assembly of mitochondrial respiratory complexes and the need to balance potential mutations in the mitochondrial DNA. The detection of damage and the ability to keep it under control is critical to fine-tune mitochondrial function to the organismal energy needs. In this review, we will analyze the multiple mechanisms that safeguard mitochondrial function in light of *in crescendo* damage. This sequence of events will include initial defense against excessive reactive oxygen species production, compensation mechanisms by the unfolded protein response (UPR^mt^), mitochondrial dynamics and elimination by mitophagy.

## INTRODUCTION

The eukaryote cell is an exceptionally complex organization of macromolecules. While initial crucial steps in the origin of eukaryote life might include the development of flexible cell surface, complex cytoskeletal organizations and heavily specialized cell compartmentalization, the ability of the cell to communicate with the external milieu has been vital for the efflorescence of specialized tissues and multicellular organisms.

The origins of mitochondria probably find their place in the engulfment and symbiotic establishment of a proteobacteria into the protoeukaryotic cell. Mitochondria act as the cellular powerhouses and play a central role in bioenergetics and metabolism of amino acids and lipids, since they host fatty acid β-oxidation, the Kreb’s cycle (also known as the tricarboxylic acid (TCA) cycle) and oxidative phosphorylation (OXPHOS) 1. However, bioenergetics is far from being the only fundamental role of mitochondria in global cell biology. Mitochondria also regulate calcium stores, lipogenesis and the production of steroid hormones 1. They can even determine cell fate via apoptotic cues. Mitochondria also play a crucial function in cellular redox homeostasis, as leakage of electrons through the electron transport chain generates reactive oxygen species (ROS) which, in controlled amounts, constitute valuable secondary messengers 2.

Nevertheless, mitochondrial physiology engages into a number of significant challenges. One of them, intrinsic to its respiratory function, is the management of O_2_ and ROS. An imbalance between ROS generation and the organismal/cellular system's ability for clearance, promotes oxidative damage to lipids, nucleic acids and proteins 2. Similarly, the mitochondrial matrix undergo massive fluxes of metabolites, such as short-chain acyl-CoAs, that can covalently bind to proteins and modify their function 3. Finally, one cannot forget that, despite mitochondria are physically delimited by two membranes, most of their proteins need to be imported and assembled in the mitochondrial compartment with exquisite stoichiometry. Therefore, there has been an evolutive pressure favoring the existence of coordinated cellular responses to mitochondrial stress and damage. This is a complex task, as the nature and extent of the damage can be largely variable. Further, hundreds of individual mitochondria can populate a cell, so there should be ways to differentiate between local damage in an individual mitochondrion from widespread mitochondrial toxicity. In the sections to come, we will explore how mitochondria respond to different and increasing types of damage, orchestrating either reparative or recycling strategies.

## MITOCHONDRIA: THE COMMUNICATING ORGANELLE 

Mitochondria have historically been viewed as relatively passive generators of the ATP that is necessary to thermodynamically drive many cellular biochemical reactions. However, mitochondria have also developed mechanisms to communicate with the rest of the cell, probably to ensure that cells do not commit to a biological event that mitochondria cannot metabolically sustain (see review 4). An early example for this concept was built in the late 1990s, upon the discovery that release of cytochrome C from the mitochondrial intermembrane space to the cytosol induced apoptosis 5.

**Figure 1:**
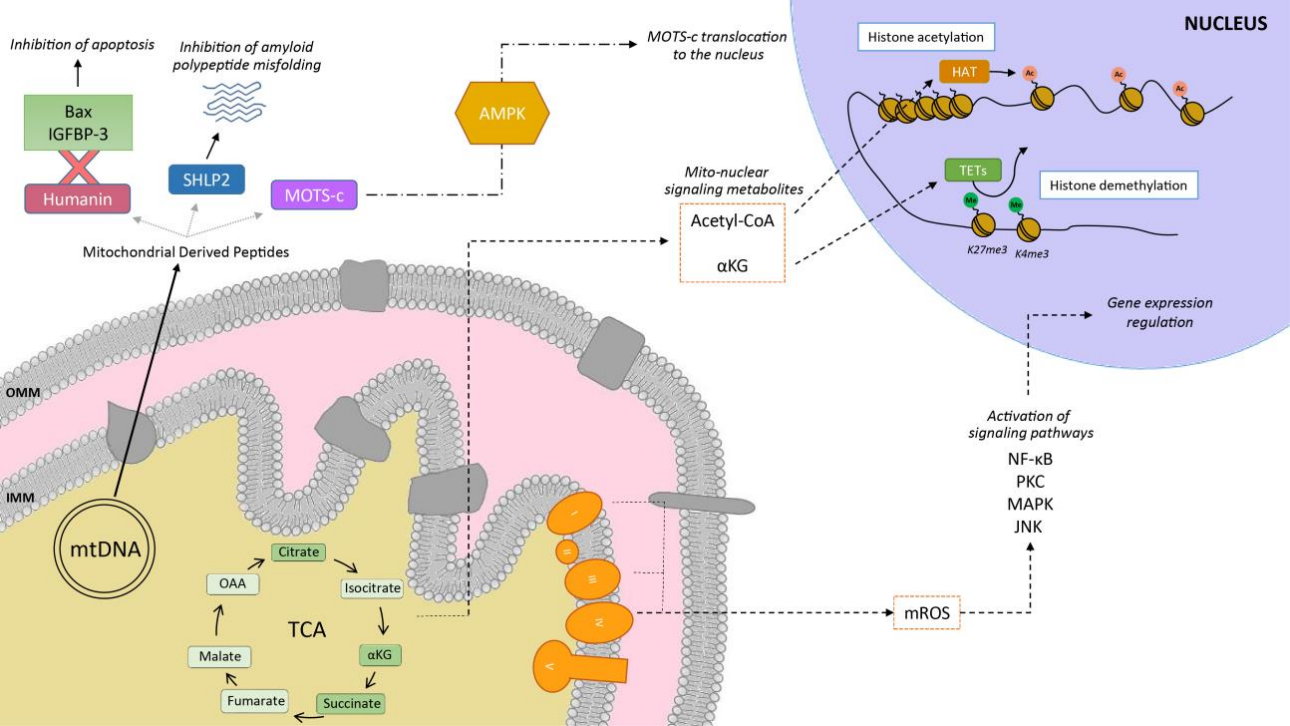
FIGURE 1: Mitochondria as communicating organelles. The figure illustrates some examples of how mitochondria communicate with the nucleus. This includes mitochondrial derived peptides (MDPs), metabolites or mitochondrial reactive oxygen species (mROS). Humanin’s cytoprotective function is mediated by its binding to Bax and IGFBP-3, preventing cellular apoptosis. Other MDPs include small humanin-like peptides (SHLPs), such as SHLP2, which specifically targets misfolded amyloid seeds to inhibit amyloid polypeptide misfolding. MOTS-c (Mitochondrial Open Reading Frame of the 12S rRNA-c) are MDPs that promote metabolic homeostasis and prevent metabolic stress by its translocation to the nucleus, provided by the metabolic regulator AMPK. Mitochondria are also one of the main sources for acetyl-CoA, which is required by the histone acetyltransferases (HATs) for histone acetylation. Acetylation of histones results in a transcriptionally active chromatin configuration that promotes gene expression. Further, the tricarboxylic acid cycle (TCA) metabolite α-ketoglutarate (αKG) is the substrate of the histone demethylase ten-eleven translocation enzymes (TETs) which demethylate the K27me3 and K4me3 histone 3 tails. Finally, mROS are generated primarily by the complexes I and III of the electron transport chain, and are then able to diffuse into the cytoplasm to activate various signaling pathways, and regulate the expression of specific genes.

Mitochondria communicate with the cellular environment through multiple molecular entities (**Figure 1**). The biochemical nature of these molecules is very broad and includes mitochondrial DNA (mtDNA) fragments, mitochondrial lipids (e.g.: cardiolipin), metabolites and small peptides (see 6 for extended review). These communication mechanisms are not necessarily linked to mitochondrial dysfunction, but used as information on multiple cues, such as nutrient fluxes or redox states. Mitochondrial derived peptides (MDPs), for example, are signaling peptides encoded by short open reading frames in the mitochondrial genome 7. MDPs contribute to a plethora of cellular pathways, by promoting cellular viability and reducing apoptosis 7,8,9. Humanin, the first discovered MDP, is a 24 amino acid peptide encoded in the 16S ribosomal RNA in the mtDNA 9, with reported neuroprotective function against amyloid β-peptide (Aβ) toxicity and Alzheimer’s disease (AD) pathology 10. Treatment of rat models with humanin inhibited the neurotoxic effect of Aβ aggregates and restored memory deficits of Aβ-induced tau hyperphosphorylation 10. In mammalian cells, humanin can bind to the apoptosis-inducing protein Bax. In response to stress, Bax can translocate from the cytosol to the mitochondrial outer membrane, where it inserts and promotes cell death through the release of cytochrome C and other apoptogenic proteins. The interaction with humanin suppressed Bax translocation to mitochondria, preventing apoptosis 11. Humanin can also be released to the blood flow 12. In this line, the interaction of humanin and circulating IGFBP-3 (IGF binding protein 3) inhibited IGFBP-3-induced cell death on human glioblastoma cells 13. Overall, this evidence demonstrates that humanin cytoprotective function is mediated by its interaction with intracellular and extracellular components 11,13. Of note, humanin’s protective role is not only restricted to mitochondria, but has also been shown to protect the endoplasmic reticulum (ER) against ER stress-induced apoptosis 14, mediated by the restoration of mitochondrial glutathione depleted by ER stress.

*In silico* analyses revealed six additional peptides encoded in the same region as humanin, named small humanin-like peptides (SHLPs) with similar function to humanin and whose levels decreased with age 9. SHLP2 specifically targets misfolded amyloid seeds to inhibit islet amyloid polypeptide (IAPP) misfolding, a critical pathogenic step in type 2 diabetes mellitus 7. In parallel to humanin and SHLPs, MOTS-c (Mitochondrial Open Reading Frame of the 12S rRNA-c) is a 16 amino acid peptide encoded within the mitochondrial 12S rRNA that promotes metabolic homeostasis and reduces obesity and insulin resistance in mice 8. HEK293 cells stably expressing MOTS-c exhibited inhibition of the folate-methionine cycle, blockade of de novo purine biosynthesis, leading to the accumulation of one of its intermediates, AICAR, and the activation of the metabolic regulator AMPK 8. Interestingly, MOTS-c was recently proved to translocate to the nucleus and regulate nuclear gene expression in an AMPK-dependent manner, promoting stress resistance against glucose restriction 15. Further, MOTS-c treated mice showed significantly enhanced glucose clearance indicative of improved skeletal muscle insulin sensitivity 8, which points out the muscle as the main target of MOTS-c. MOTS-c treatment in mice also prevented age-dependent and high fat diet induced insulin resistance and obesity 8. These observations suggest that mitochondria actively regulate cellular cues by releasing peptides encoded within their own genome.

Metabolites released from the mitochondria to the nucleus also act as retrograde signaling systems, and many of them constitute substrates for chromatin-modifying enzymes (**Figure 1**). This allows coupling chromatin-dependent gene regulation with the metabolic state of the cell 16. For instance, acetyl-CoA is an intermediary metabolite that participates in the mitochondrial TCA cycle by interacting with oxaloacetate to produce citrate, but also has a signaling role by the acetylation of proteins. It is generated from acetate, citrate and pyruvate, and also by the breakdown of both carbohydrates (glycolysis) and lipids (β-oxidation). Acetyl-CoA production is essential for the activity of histone acetyltransferases (HATs), enzymes responsible of acetylating histone lysine residues. Acetylation of histones decreases the interaction between histones and DNA, giving rise to a transcriptionally active chromatin configuration that promotes gene expression. Initial observations on the interplay between the metabolic state and histone acetylation response to growth were made in yeast. In yeast, one pathway to produce acetyl-CoA relies on acetyl-CoA synthetases Acs1p and Acs2p, which catalyze the ligation of acetate and CoA. Acs2p yeast mutants exhibited global histone deacetylation, correlated to broad decreases in gene expression and growth defects 17. In a complementary study, elevated glucose levels in yeast resulted in fueled production of acetyl-CoA, promoting the activity of the transcriptional coactivator complex SAGA (Spt-Ada-Gcn5-Acetyltransferase) 18, to acetylate histones specifically at the genes responsible of cell growth 19. Hence, a yeast cell coordinates its growth with the production of acetyl-CoA, which is indicative of its metabolic and nutritional state 19. These observations of metabolic cues directly altering histone acetylation have also been extended to mammalian cells. Glucose is the major source for mammalian cells and it can be used by ATP-citrate lyase (ACL), the enzyme that converts glucose-derived citrate into acetyl-CoA. RNA-induced silencing of ACL led to the decrease in global histone acetylation in response to growth factor stimulation, while it also resulted in impaired differentiation in adipocytes 20. ACSS2 (the mammalian equivalent of Acs2p) is highly expressed in the mouse hippocampus, the area in charge of memory consolidation. Attenuated ACC2 expression in adult mice showed impaired long-term spatial memory, correlated to a defective upregulation of immediate-early memory genes 21. Acetyl-CoA generation via ACSS2 establishes, thus, a connection between cellular metabolism, epigenetic modifications and, in the above case, neuronal plasticity 21. Similarly, another key chromatin modification that is strongly interconnected with metabolism is methylation. In this sense, some demethylases, such as the ten-eleven translocation (TET) methylcytosine hydroxylases require α-ketoglutarate (α-KG) as an essential co-substrate 22. α-KG is a TCA metabolite that functions as a co-substrate for 2-oxoglutarate-dependent dioxygenases, which catalyze hydroxylation reactions on various types of substrates. High levels of α-KG promote TET-dependent DNA demethylation of K27me3 and K4me3 histone 3 tails 23, again pointing towards an intimate link between metabolism and DNA demethylation.

Mitochondria are also an important source for ROS. ROS molecules have long been known as being damaging and pernicious agents to the cell, resulting in oxidative stress (see section 2, below). However, ROS play a critical role as signaling molecules to maintain physiological functions. In this regard, superoxide anions and hydrogen peroxide were probed to activate signaling pathways controlled by tyrosine phosphorylation such as NF-κB, PKC, MAPK or JNK 24,25 (**Figure 1**). ROS react with the redox-sensitive cysteine residues of tyrosine phosphatases leading to their transient inactivation, which favors unopposed kinase activity 26,27. Nowadays, ROS have been demonstrated to contribute in many physiological events including adaptation to hypoxia and physical activity, regulation of autophagy, immunity, differentiation and longevity (detailed in 26). For instance, ROS formation and low-level stress due to reduced glucose availability (calorie restriction) was proposed to culminate in stress resistance 28 and was shown to extend life span in *C. elegans* 29. This was one of the founding observations for the concept of mitochondrial hormesis or mitohormesis, in which mitochondrial stresses rapidly activate cytosolic signaling pathways that ultimately alter nuclear gene expression aimed to strengthen the defense towards the initial stress 30,31. In this sense, mitochondrial ROS production can be observed as a mitohormetic signal. Hence, there is no linearity between ROS production and cellular toxicity. Nevertheless, given that excessive ROS production can be damaging to the cell, mitochondrial ROS levels are tightly regulated by multiple systems in order to ensure their ability to participate in physiological cell signaling while preserving cell homeostasis, as described in our next chapter.

## OXIDATIVE STRESS

The integration of an α-proteobacterium within an ancient host cell around 1.45 billion years ago was key for the survival and replication of the host in an environment with increasing oxygen (O_2_) levels. This proteobactium helped in the removal of the O_2_ that was being diffused inside the host cell from the environment 32,33. The coupling of O_2_ consumption to ATP synthesis allowed the sustainability of eukaryotic cellular bioenergetics as we know them today. In addition, aerobic metabolism spurred the generation of new metabolites such as steroids, alkaloids and isoflavonoids 34,35.

O_2_ is nowadays not only linked to ATP synthesis through the mitochondrial electron transport chain (ETC), but also to the remodeling of protein structure and function, for example, via post-translational modifications 36. Nevertheless, some of the metabolic products of the oxidative phosphorylation remain a threat to cell survival when their production and removal are not properly balanced. The ETC is one of the main producers of ROS, a wide name for a constellation of oxygen anion forms that include superoxide (O_2_^−^), hydrogen peroxide (H_2_O_2_) and the hydroxyl free radical (OH-). O_2_^−^ causes the formation of other reactive species: it can directly produce OH- or indirectly through the dismutation of H_2_O_2_. H_2_O_2_ has low reactivity, but high penetrability in cell membranes and when it accumulates, it is highly toxic to cells 37,38. Also, H_2_O_2_ can be converted to OH- in the presence of Fe^2+^ 39. OH- is the most reactive and dangerous form of oxygen, as it can react with all biological macromolecules 38,40.

Excessive ROS production or an ineffective antioxidant response results in oxidative stress 41, promoting mitochondrial dysfunction and affecting cell viability by damaging nucleic acids, proteins and lipids. In this regard, ROS accumulation can lead to DNA base modification, DNA strand breaks, inter- and intra-strand crosslinks and DNA-protein crosslink that ultimately affect genomic structure and stability 42. Similarly, changes in protein structure and function can arise from the oxidation of cysteine residues, which are intrinsically vulnerable to oxidative stress because of the highly reactive nucleophilic thiol moiety 43. Lipid membranes promote the formation of lipid radicals when exposed to free radicals, leading to the most devastating effect of oxidative stress, which is lipid peroxidation and altered membrane permeability and stability, ultimately compromising cellular compartmentalization and overall function 44. Not surprisingly, the macromolecular damage caused by increased oxidative stress has been linked to multiple pathologies such as atherosclerosis, diabetes, cancer and chronic inflammatory processes, as well as age-related physiological deterioration 37,45,46,47,48.

**Figure 2:**
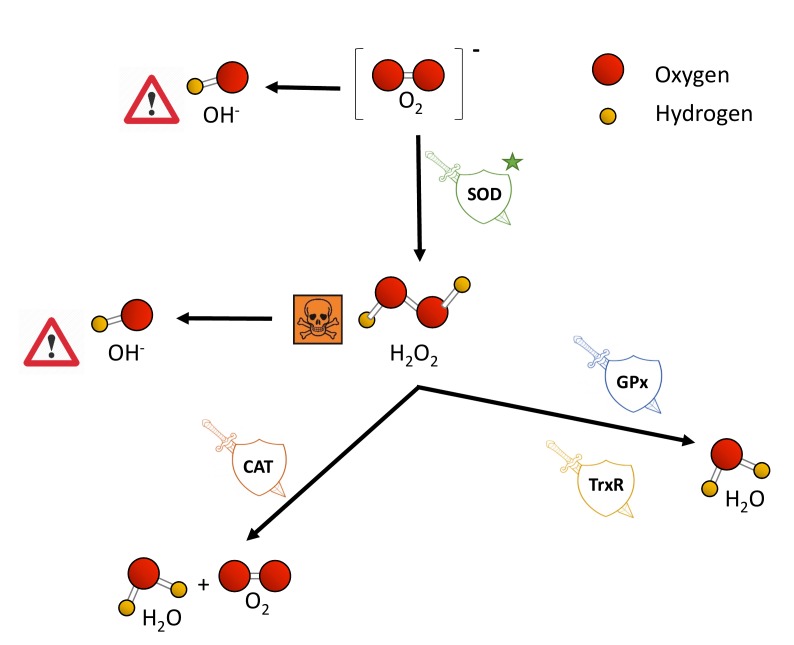
FIGURE 2: Toxic ROS generation and enzymatic antioxidant defenses. Cells encompass a group of enzymes which help counterbalancing the potential detrimental effects of ROS production upon toxic levels. O_2_^-^, generated as a by-product from the electron transport chain in mitochondria, is converted to H_2_O_2_ by superoxide dismutases (SOD), which are the first and major line of defense against ROS. H_2_O_2 _accumulation is highly toxic for cells, and it is subsequently transformed to H_2_O and molecular oxygen by the catalase enzyme (CAT). Glutathione peroxidases (GPx) and thioredoxin reductases (TrxR) also aid in the conversion from H_2_O_2_ to water. Independently, H_2_O_2 _is also converted to OH^-^.

Eukaryotic cells harbor complex antioxidant strategies to protect against an uncontrolled increase in free radicals. This includes both enzymatic and non-enzymatic mechanisms (i.e. vitamins). The enzymatic response is primarily mediated by superoxide dismutases (SOD), catalases, thioredoxin reductases or glutathione peroxidases 49. SOD enzymes catalyze the conversion of O_2_^−^ to H_2_O_2_ and O_2_, and they are considered as one of the most powerful antioxidant agents in cells (**Figure 2**) 38. Various forms of SOD enzymes exist, that differ in their cellular localization and the metal cofactor used for their catalytic activity 50. SOD1 is a soluble Cu/Zn enzyme that is mainly present in the cytosol, although a small percentage (approx. 3%) also exists in the intermembrane space of mitochondria 51. Upon increased levels of H_2_O_2_, SOD1 can translocate to the nucleus, where it binds to DNA promoters and favors the expression of oxidative resistance and repair genes 52. MnSOD (encoded by the SOD2 gene) has been described to be located exclusively in mitochondria 53, being the primary defense against mitochondrial oxidative stress. In this sense, SOD2 deletion has been linked to defective pancreatic β-cell secretory capacity 54, as well as cancer progression in a tissue-specific manner 55,56. SOD3 is a secretory extracellular Cu/ZnSOD which is expressed highly in selected tissues, including the gastrointestinal tract, blood vessels, lung or kidneys 57. Once released, SOD3 binds to the surface of endothelial cells rich in sulfated polysaccharides such as heparin and heparan sulfate, and this helps endothelial cell function by protecting from oxidant-mediated damage, inflammation, and interstitial fibrosis in lung 58,59.

Catalases (CAT) can break down H_2_O_2_ into water and molecular oxygen by utilizing iron or manganese as a cofactor, thereby completing the detoxification initiated by SOD 60. CAT enzymes are predominantly found in peroxisomes but absent in mitochondria of mammalian cells 61,62. In this case, the conversion of H_2_O_2_ to H_2_O and O_2_ is carried out by another type of enzymes known as glutathione peroxidases (GPx), which use a selenium intermediate and glutathione as substrates 63. GPx play also a crucial role breaking lipid peroxides to their alcohols, which protects cells from oxidative stress 64,65. The expression of individual GPx is tissue-specific and has been classified into eight groups 66,67. For example, GPx1 is the most abundant form and is present in all cells; GPx2 is exclusively expressed in the gastrointestinal track, providing a barrier against hydroperoxides produced in the diet, while plasma GPx (GPx3) is directed to extracellular compartments and is mainly expressed in tissues in contact with body fluids (i.e, kidney) (for a detailed review, see 68). While both GPx and catalases use H_2_O_2_, it has been proposed that the glutathione redox cycle is a source of protection against mild oxidative stress as compared to CAT, which protect against severe oxidative stress 69.

Thioredoxin reductases (TrxRs) are enzymes characterized by the redox activity of its flavin adenine nucleotide (FAD) group, which helps reducing thioredoxins (Trxs) by the oxidation of NADPH to NADP^+^ 70. Trx, once reduced, supplies electrons to enzymes such as peroxiredoxins or Trx peroxidases, which regulate the conversion of H_2_O_2_ into water 71,72,73. Mammalian cells contain two TrxRs, the cytosolic TrxR1 and the mitochondrial TrxR2. Apart from protection against oxidant stress, some of the biological functions of TrxRs include cell growth, promoting transcription factor activity, ascorbate recycling or tumor resistance 74. Both TrxRs and GPx have been demonstrated to also protect against nitrosative stress, concretely nitrosothiols and peroxynitrite 75.

The expression of ROS metabolizing enzymes can be directly controlled by oxidative stress. Primary responses to ROS are modulated by the cooperation of the NF-κB, AP1 and MAPK pathways. When these responses are not sufficient to counteract the increase in ROS, the NRF2/KEAP cascade is activated to induce antioxidant defenses and minimize oxidative damage 49. The mitochondrial complex I, although being one of the main generators of O_2_^−^, also contributes to the antioxidant response by inducing the NRF2/KEAP pathway mediated by fumarate accumulation and ERK5 activation 76.

Overexpression or knockout of the ROS metabolizing enzymes has helped in the understanding of the function and activity of the different groups of antioxidant enzymes, as well as how their dysregulation can lead to disease. For instance, SOD3 ablation in adult mice caused increased lung superoxide content, inflammation, respiratory acidosis and reached 85% mortality within six days 77. SOD3 is usually repressed in the tumor microenvironment, but re-expression of the enzyme in tumor-associated endothelial cells improved tumor perfusion and selective chemotherapy delivery 78. There are many other SOD enzymes involved in disease, as is the case of SOD1, which is linked to inherited amyotrophic lateral sclerosis (ALS) 79. We find more examples in which re-introduction of a ROS detoxification enzyme leads to disease amelioration. The overexpression of catalase in a breast cancer model increased the sensitivity of the tumor cells to paclitaxel, etoposide and arsenic trioxide, redox-based chemotherapeutic drugs 80. Catalase deficiency has also been linked to the development of type 2 diabetes mellitus 81, cardiac aging and hypertension 82, or increased DNA damage during UVB irradiation 83. In contrast, catalase overexpression has been demonstrated to protect the mitochondria of insulin-secreting cells against ROS toxicity and cytokine-mediated cell destruction 84. However, the presence of ROS detoxification enzymes is not always correlated to a positive outcome. In this regard, Gpx1 expression and activity have been shown to increase in mouse livers after the induction of hepatitis. Lee et al. (2016) reported that, in fact, Gpx1 KO mice presented an attenuation of liver injury by inhibiting cytokine production 85. These disparate observations testify for a clear role of ROS detoxifying enzymes in multiple pathophysiological settings and emphasize how different ROS detoxification enzymes may play opposite roles depending on the level of oxidative stress and in the type of tissue.

## THE UNFOLDED PROTEIN RESPONSE

To guarantee the appropriate folding, assembly and turnover of proteins under normal and stress conditions, cells modulate the levels of proteases and chaperones involved in protein quality control. In eukaryotes, the cytosol, ER and mitochondria are all exposed to nascent polypeptides, thus each compartment requires specific protein-folding machinery and responds differently upon unfolded polypeptides, signaling to the nucleus to induce the expression of organelle-specific chaperones. The unfolded protein response (UPR) encompasses, thus, a collection of signaling pathways that evolved to restore an efficient protein-folding environment. The mitochondrial UPR (UPR^mt^), the endoplasmic reticulum UPR (UPR^ER^) and the cytosolic heat-shock response (HSR), exist as quality control mechanisms against proteostatic stress cues that put at risk cellular homeostasis, and that can lead, ultimately, to cell death and apoptosis.

### UPR^mt^

One of the first hints to the existence of a mitochondrial UPR was provided by the Hoogenraad laboratory in a simple yet pioneering experiment overexpressing OTC-Δ - a mutant form of the mitochondrial matrix protein Ornithine transcarbamylase - as a way to promote protein misfolding and aggregation. This artificial paradigm to induce variations in the stoichiometry of the mitochondrial- and nuclear-encoded proteins in the mitochondrial matrix led to the increased expression of HSP60 and CLPP (ATP-dependent caseinolytic protease proteolytic subunit) in cultured mammalian cells 86,87. Co-immunoprecipitation analysis revealed that both HSP60 and CLPP were stably associated with OTC-Δ, but not with the wild type OTC, suggesting a possible role of these stress-induced proteins in resolving misfolded proteins 87. Furthermore, this response was shown to be organelle-specific, as the ER- and cytoplasmic-specific chaperones were not affected 86,87. Hence, it was established that the accumulation of misfolded proteins in mitochondria produces a mitochondrial stress response, known as the mitochondrial unfolded protein response (UPR^mt^), characterized by the upregulation of nuclear genes encoding mitochondrial molecular chaperones and proteases, in order to ensure the functional integrity of the mitochondrial proteome 87. However, it is not yet clear if the induction of the UPR^mt^ *per se* is enough to reduce the abundance or aggregation of misfolded proteins. Similarly, it is not clear whether the removal of these aggregates is required for the potential recovery of mitochondrial function after UPR^mt^.

Some of the factors triggering the UPR^mt^ include mtDNA depletion, impaired mitochondrial protein quality control or OXPHOS dysregulations. These defects influence one another, as OXPHOS or mitochondrial proteostasis perturbations reduce the rate of protein import inside mitochondria by increasing the inner mitochondrial membrane (IMM) proton permeability, which consequently dissipates the proton gradient and causes precursor proteins to remain outside the mitochondria 88,89.

The mitochondrial genome encodes 13 proteins that are constituents of the OXPHOS respiratory complexes, but the rest of the mitochondrial proteome is encoded in nuclear genes, synthetized in the cytosol and imported to the different mitochondrial compartments via the TOM (translocase of the outer membrane) and TIM (translocase of the inner membrane) channels 90,91. Because the majority of proteins in mitochondria are synthesized by cytosolic ribosomes, proper protein translocation inside the organelle becomes vital for its function. Protein import and folding into the mitochondrial intermembrane space (IMS) relies on disulphide bond formation, and this is carried out by the mitochondrial disulphide relay machinery, using O_2_ in the process 92. During stress conditions, such as hypoxia, uncontrolled ROS production or mismatched rates of protein import, mitochondria have developed protein folding assistance mechanisms to preserve proteostasis, as described below.

**Figure 3:**
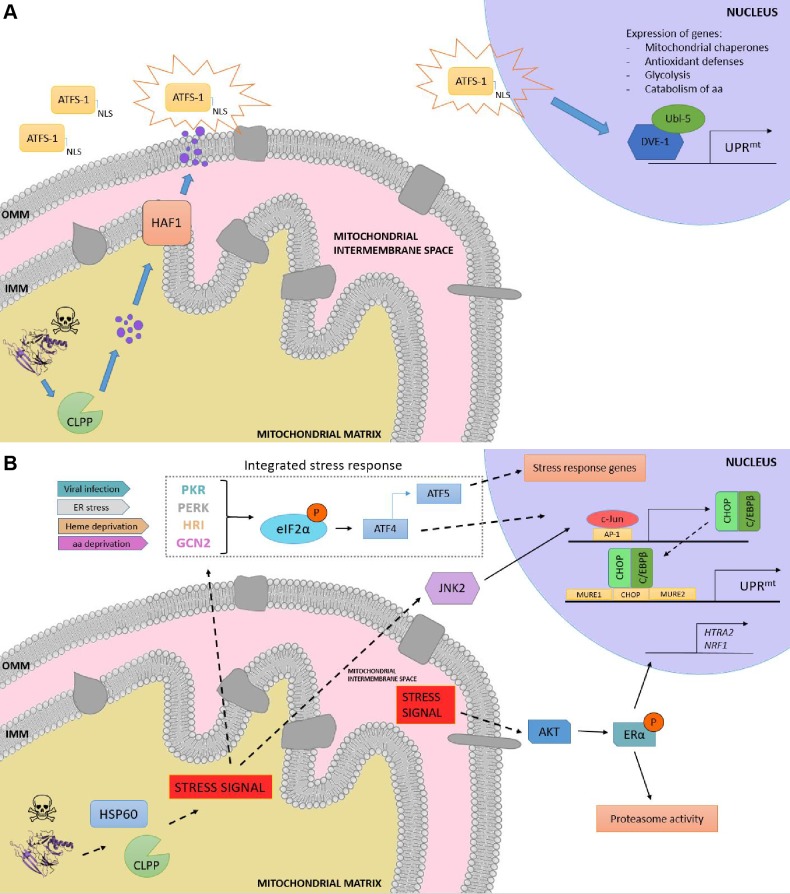
FIGURE 3: Overview of the UPR^mt^ in worms and mammals. (A) UPR^mt^ in *C. elegans.* When the capability of the mitochondria to process unfolded proteins is compromised, the mitochondrial unfolded protein response (UPR^mt^) is activated. Upon mitochondrial stress, the protease CLPP degrades the excess of unfolded proteins, and the resulting fragments are moved across the inner mitochondrial membrane (IMM) to the intermembrane space, where they diffuse through passive diffusion to the cytoplasm. ATSF-1 import inside mitochondria is prevented, and its activation upon the sensing of the degraded peptides shuttles ATFS-1 to the nucleus, granted by the nuclear localization sequence (NLS). **(B) Retrograde signaling in mammals upon proteotoxic stress**. The excess of unfolded proteins is sensed by quality control proteases and chaperones such as HSP60 and CLPP, and the stress signal is processed via two different pathways, the activation of the CHOP gene via the JNK pathway and the induction of the integrated stress response (ISR), respectively. On the one hand, JNK2 processes the stress signal from the mitochondrial matrix to activate the transcription factor c-Jun, which binds to AP-1 elements and induces the expression of CHOP and C/EBPβ. The CHOP-C/EBPβ complex then binds to the CHOP element, which is flanked by two mitochondrial unfolded protein response elements (MURE1 and MURE2), activating the transcription of UPR^mt^ -responsive genes. On the other hand, viral infection, ER stress, heme deprivation and amino acid insufficiency activate PRK, PERK, HRI and GCN2, respectively, giving rise to the phosphorylation of eIF2α, the core of the ISR. This leads to the preferential translation of ISR-specific mRNAs, such as ATF4, the main effector of the ISR. ATF4 enhances the transcription of ATF5, leading consequently to the transcription of target genes. Moreover, a matrix-independent IMS- specific UPR^mt ^has also been reported. Stress signals coming from the accumulation of unfolded proteins in the IMS leads to the AKT-mediated phosphorylation of ERα, resulting in the expression of HTRA2, NRF1, and an increase in the activity of the proteasome.

Although initially discovered in mammals 86, the elucidation of the UPR^mt^ molecular mechanism has been mostly characterized in *C. elegans *due to the relative ease of using this organism to perform genetic screenings. In worms, the response is characterized by the action of regulators including the mitochondrial matrix protease CLPP, the ATP-binding cassette (ABC) transporter HAF-1 and the basic leucine zipper (bZIP) transcription factor ATFS-1. Briefly, upon mitochondrial proteostatic stress, CLPP proteolytic activity degrades misfolded proteins in the mitochondrial matrix, and the small peptides are then transferred across the IMM via HAF-1. Afterwards, these peptides are moved through the outer mitochondrial membrane (OMM) by passive diffusion to the cytosol (**Figure 3a**). This activates ATFS-1, which translocates to the nucleus thanks to a nuclear localization sequence (NLS) and activates the ubiquitin-like protein UBL-5 to form a complex with the transcription factor DVE-1 93. ATSF-1 and DVE-1/UBL-5 then cooperatively regulate the transcription of mitochondrial chaperones including HSP60 and mtHSP70 94*. *Recent studies also demonstrate that chromatin is specifically remodelled during mitochondrial dysfunction to activate the UPR^mt -^ responsive genes. The histone methyltransferase MET2 in concert with LIN65 promote global chromatin condensation, while the histone demethylases JMJD-3.1 and JMJD-1.2 maintain the promoters of UPR^mt^-induced genes in a transcriptionally competent state, and this structure is further stabilized by the DVE-1/UBL-5 complex to facilitate ATFS-1 access to chaperone promoters 95.

Other UPR^mt^ response gene sets encode as well for mitochondrial proteases, ROS detoxification enzymes, and mitochondrial protein import components 96, aimed to restore mitochondrial homeostasis. Furthermore, metabolic adaptations inevitably take place during mitochondrial stress, as ATFS-1 also promotes the transcription of glycolysis genes, which aids sustaining ATP levels 97 and the mevalonate pathway 98, which feeds the synthesis of cholesterol, heme groups, coenzyme Q10 and steroid hormones 99. Once mitochondrial function is restored, ATFS-1 is imported again into mitochondria, where it is degraded by the Lon protease. Thus, ATFS-1 accumulation on the cytosol upon impaired protein import efficiency acts as both a proteostatic sensor and a mitochondria-to-nucleus signaling mechanism. It should be noted, however, that the set of genes upregulated by the UPR^mt^ in *C. elegans* is different to that of mammalian cells. This emphasizes that the mechanisms of proteostatic regulation differ between organisms and that equivalences between models should be taken with some caution. For instance, although some of the components of the pathway, such as the mitochondrial chaperones and the quality control protease CLPP were shown to be conserved from *C. elegans* to mammals, the closest mammalian homologs of DVE-1, SATB1 and SATB2, are unable to stimulate the UPR^mt^ 100. In addition, mammals possess several signalling paths and transcriptional regulators that might redundantly influence the response.

In mammals, the UPR^mt^ is mediated by the CHOP- C/EBPβ system 87,101, in which the sensing of unfolded proteins is transmitted by a retrograde signaling to the nucleus, leading to the activation of the CHOP gene. The transcription factor c-Jun is activated via JNK2 and binds to the CHOP and C/EBPβ promoters, respectively, thanks to the presence of the AP-1 binding site (**Figure 3b**). Once expressed, the CHOP- C/EBPβ system binds to specific gene promoters, characterized by the presence of the CHOP binding site and two mitochondrial unfolded protein response elements (MURE1 and MURE2), promoting the transcription of UPR^mt^ -responsive genes. The bioinformatics approach by Aldridge *et al*. (2007) identified seven genes UPR^mt^ -responsive, including YMEL1L1, mitochondrial thioredoxin 2 (Trx2), NDUFB2 (subunit of complex I) or CLPP 101. Nevertheless, CHOP specificity in the UPR^mt ^is questionable, as its promoter contains both an UPR^ER^ and an UPR^mt^ response element 102. Furthermore, CHOP can also be induced by other mitochondrial stresses unrelated to protein folding. Hence, the induction of CHOP should not be taken as a direct readout of UPR^mt^ 87,103. Interestingly, some studies point out to a matrix-specific UPR^mt^ that senses excess of unfolded proteins in the IMS and that is independent of CHOP 104. This was demonstrated through the overexpression of a mutant form of endonuclease G (EndoG), an IMS-specific nuclease, that lead to the formation of aggregates in the mitochondrial IMS, which results in IMS stress 104 and ROS overproduction. ROS-dependent AKT phosphorylation activated the estrogen receptor α (ERα) 105,106, which promotes the expression of the IMS protease HTRA2, the nuclear respiratory factor NRF1, and the activity of the proteasome 107.

Induction of the integrated stress response (ISR) has also been reported to coordinate the UPR^mt ^in mammals, in a mechanism dependent on the phosphorylation of the eukaryotic translation initiation factor 2 (eIF2α) 108,109. Although eIF2α phosphorylation leads to inhibition of global protein synthesis, specific mRNAs can bypass this limitation if they have small open reading frames in their 5’UTR (uORFs), which is the case for the activating transcription factor 4 (ATF4), CHOP and the functional ortholog of ATFS-1, ATF5. ATF4 has been described to enhance ATF5 expression 110, which can contribute to transcriptional adaptations to mitochondrial stress 111. In a complementary fashion to that of HAF-1 and ATFS-1, eIF2α can be activated by four different forms of cellular stress: PERK (ER stress), PRK (presence of double-stranded RNA), HRI (heme deficiency) and GCN-2 (amino acid starvation) 108. Constitutive expression of a phosphomimetic S49D mutation in eIF2α conferred dramatic effects, impairing growth, oxidative metabolism and reproduction in transgenic worms 112.

Moreover, several mitochondrial stress responses (MSRs) are induced by other factors different to protein misfolding and that can trigger the ISR. For instance, Tyynismaa *et al*. (2010) developed a mouse model carrying a mutation in TWINKLE - a mtDNA helicase - that lead to the accumulation of mtDNA deletion, resulting in respiratory chain deficiency and the induction of the MSR 113. Interestingly, global gene expression patterns showed induction of pathways involved in amino acid starvation and lipid metabolism regulation, particularly Fgf21 114. Fgf21 was upregulated in the skeletal muscle of the mutant mice, correlated to small adipocyte size, low fat content in the liver and resistance to high-fat diet 114. This study exemplifies how mitochondrial stress responses, which are commonly activated upon pathogenesis of mitochondrial disorders, can lead to reprogrammed cellular metabolism. Further, the recent work by Quirós *et al*. (2017) showed the overlapping between the MSR and the ISR via the specific activation of ATF4, which resulted in the upregulation of pathways related to biosynthesis of amino acids, in particular serine 115. The authors discussed that this increase in serine might be used to promote the synthesis of lipids and phospholipids, as these pathways were the most highly induced upon mitochondrial stress. All the above studies point out to the conclusion that mitochondrial stress responses might share effector pathways, irrespectively of the nature of the stress. Further work will be needed to fully understand how the specificity and threshold of the damage is determined.

Mitochondrial function is also altered by its communication with other cellular organelles, besides the nucleus. Mitochondria physical interaction with the ER has many functional implications, including calcium exchange or lipid transfer, but also targets the ER as one of the first sites when mitochondrial function is disrupted. The association between defective mitochondria and the ER has been demonstrated to increase ER stress, resulting in incorrect protein folding 116, source of neurodegenerative disorders such as Parkinson’s disease.

### UPR^ER^

The accumulation of toxic, unfolded proteins in the ER leads to ER stress and the activation of the ER unfolded protein response (UPR^ER^). The UPR^ER ^was the first stress response identified 117,118, and because of this it is still often referred simply as the UPR. Different mechanisms seek to restore normal ER function by increased expression of ER-specific chaperones. In mammals, the IRE1, PERK and ATF6 mechanisms monitor correct ER-specific protein folding function through direct interaction with BiP (ER chaperone-binding immunoglobulin protein). These signaling pathways activate transcription and translational mechanisms that reduce global protein synthesis, increase ER protein-folding capacity, and promote the degradation of misfolded proteins 119. Variations exist in the key players of the UPR between organisms, which correlated with the level of evolution of their organelles. IRE1 - considered the most ancient UPR pathway - is present in budding yeast, plants, fungi and metazoans. Protozoans, however, do not have orthologues of IRE1. The activation mechanism of IRE1 has not been yet fully elucidated, although recent studies point out that post-translational modifications may determine its functionality, such as phosphorylation at Ser729 120. Although the activation mechanisms for IRE1, PERK and ATF6 differ, the three pathways can communicate with each other, as illustrated by the regulatory feedback of the XBP1-ATF6 axis 121,122, or by the enhanced IRE1α-XBP1 signaling via PERK 123, which may enable cells to cope with various types and intensities of ER stress. Moreover, Bax and Bcl2 - which oligomerize in the OMM upon activation - can also localize to the ER upon ER stress. As compared to mitochondria, Bak depletes the ER of Ca^+2^ and induces Caspase12 cleavage and consequently apoptosis 124. These results point out to a co-regulation between the mitochondrial- and the ER-UPR. One of the critical connections between the ER and mitochondrial function is established by the direct connection between both organelles. Mitochondria are spatially and functionally organized in a network in close contact with the ER 125, and this contributes to mitochondrial uptake of Ca^+2^ released from the ER by inositol-1,4,5-triphosphate (InsP_3_) and also sustains lipid biosynthesis, which occurs at sites of ER membranes attached to mitochondria that contain phospholipid and glycosphingolipid biosynthetic enzymes 126,127.

Multiple models of mitochondrial dysfunction are characterized by ER stress. Elevated levels of free Ca^+2^ due to mitochondrial dysfunction have been linked to the induction of ER stress in a p38 MAPK-dependent manner, giving rise to aberrant insulin signaling and hepatic gluconeogenesis 128. Ablation of Mfn2 - a key component in the mitochondrial fusion machinery localized in the OMM - triggers all three UPR^ER^ and determines cell fate 129. ER stress impacts, reciprocally, over mitochondrial proteostasis, as it is the case of the cytochrome C oxidase (COX) expression and assembly. Hori *et al*. (2002) demonstrated that suppression of protein synthesis due to ER stress had an effect on the assembly of the COX complex by disruption of the synthesis of COX subunits and ATP-dependent enzymes 130. Moreover, suppression of PERK leads to the upregulation of the mitochondrial matrix proteins Lon, mtHsp70 and Yme1, and this aids proteostasis capacity. Physical and functional interactions between mitochondria and the ER are, thus, essential for cellular function and survival. Disruptions of ER-mitochondria tethering have been reported in human neurodegenerative disorders 131, which emphasizes that, rather than considering the two organelles separately, a better understanding of human pathologies can derive from studying the alterations in their crosstalk.

### HSF

The cytosolic UPR is mediated mainly by the heat-shock factor (HSF) family of proteins, maintaining protein-folding homeostasis in the cytosol. In eukaryotes, ribosome-associated chaperones such as NAC (nascent chain associated complex) and RAC (ribosome associated complex) interact with the ribosome and bind to hydrophobic elements of newly synthesized polypeptides, facilitating folding or polypeptide transfer to downstream chaperones 132. Stress-inducible cytosolic Hsp70 functions with Hsp40 to assist in the folding of 20% of newly synthesised polypeptides in an ATP-dependent manner 133,134, and Hsp70 also helps in protein trafficking and degradation of misfolded proteins 135. Finally, proteins that are unable to fold by chaperones are transferred to the chaperonin cages. Encapsulation has been suggested to accelerate the folding rate of the unfolded peptides over spontaneous folding. The TRiC (T-complex protein-1 Ring Complex; also called CCT) eukaryote chaperonin system promotes ATP-dependent folding of approximately 10% of the eukaryotic proteome 136, even though it is still not yet fully understood how TRiC is capable of discerning between non-folded substrates and the folded counterparts. Nuclear magnetic resonance (NMR), crosslinking-mass spectrometry and modeling approaches suggest that recognition codes in the polypeptide enable substrate recognition by TRiC 137. Significantly, TRiC chaperonins have been linked to numerous pathologies, such as neuropathies 138, oncogenesis 139, or age-related diseases 140,141. A connection between the cytosolic UPR and mitochondrial function has recently been described by means of the heat shock factor 1 (HSF1), which is a quality control regulator with a role in systemic energy sensing and metabolic adaptation to nutrient availability 142. The article by Qiao *et al*. (2017) reported that, in the absence of HSF1, the levels of NAD^+^ and ATP are not sustained in hepatic cells, and this increased protein acetylation and impaired mitochondrial integrity. Furthermore, the activity of HSF1 upon low levels of mitochondrial stress regulates cytoplasmic proteostasis and healthspan in worms 143, emphasizing - as it occurred for the UPR^ER ^- the intricate regulation between the cytosolic UPR and mitochondrial function.

Damaged mitochondria that are not salvageable by the UPR can be identified and specifically degraded through a process called mitophagy (originally, mitophagocytosis). This process helps eliminate the impaired mitochondria that could harm the rest of the functional network, and will be discussed in the next section.

## MITOCHONDRIAL DYNAMICS AND MITOPHAGY: EASY COME, EASY GO

Mitochondrial dynamics are the repetitive cycles of fission and fusion of the mitochondrial network. These are highly orchestrated events influenced by a variety of physiological and environmental cues. For instance, nutrient supply: nutrient overload is linked to a fragmentation of the mitochondrial network, while mitochondria elongate under starvation 144. Furthermore, prior to mitosis, mitochondria go through fission events to guarantee an equal distribution to the daughter cells. The balance between fission and fusion activities will determine the architecture of the mitochondrial network and influences multiple mitochondrial functions, including respiratory coupling, calcium buffering or apoptosis. In this regard, the disruption of mitochondrial dynamics can give rise to a wide range of health and metabolic diseases, including diabetes and obesity 145,146, as well as heart failure 147, Alzheimer disease 148,149,150,151, Parkinson disease 152,153, and age-related physiological decline 154,155.

Fission and fusion events act as a quality control mechanism by removing defective mitochondria or by selecting the organelles with the optimal matrix metabolites, intact mtDNA copies and mitochondrial membrane components. This interplay between mitochondrial dynamics and mitophagy - the selective removal of mitochondria by the autophagic machinery - assures the homeostasis of the cell. When one of these elements fails, dysfunctional mitochondria are not properly removed from the cellular pool, generally leading to higher amounts of ROS production and increased susceptibility to release cytochrome c and apoptosis-inducing factor (AIF) 156,157.

Generally, mitochondrial fragmentation precedes mitophagy as mitochondria that are smaller are easier to be engulfed by the autophagosomes (APs) 158,159,160,161,162. APs fuse with lysosomes and acquire acid hydrolases, which finally degrade the engulfed mitochondria. Previous studies indicate that fission is the main trigger of membrane depolarization in mitochondria. Twig *et al*. (2008) showed, by measuring the bioenergetic profile during fusion/fission events in COS7 and INS1 cells, that mitochondria tend to maintain a stable membrane potential ΔΨ_m_ (within ( 2.7 mV) 163. However, fission events generate large changes in ΔΨ_m_ and, in the majority of the cases, one daughter mitochondria will be depolarized while the other will hyperpolarize (ΔΨ_m _difference > 5 mV), resulting in asymmetric daughter mitochondria. Depolarization below a certain ΔΨ_m_ is associated with impaired mitochondrial function and acts as the trigger for mitophagy 163,164.

The perspective of mitochondrial fission acting upstream of mitophagy was validated by genetic manipulation of the pro-fission proteins Fis1 (Fission 1 protein) and Drp1 (Dynamin-related protein 1). The mammalian Drp1 is a GTPase that localizes mostly in the cytosol, but that can cycle on and off the OMM, where it can be docked by a set of different proteins, such as Fis1, the mitochondrial fission factor (Mff) or MiD49/51 165. Once recruited to the OMM, Drp1 oligomerizes and constricts the mitochondria, which gives rise to two daughter organelles 166. Knockdown of Fis1 (by siRNA) or overexpression of a dominant negative Drp1 isoform (Drp1 K38A) resulted in the reduction of the number of mitochondrial-containing autophagosomes, but not in the total number of lysosomes 163,167. As degradation of dysfunctional mitochondria was impaired, levels of mitochondrial protein oxidation were increased with no significant ROS overproduction. Nevertheless, some studies indicate that mitophagy can also occur independently of Drp1 activity upon proteotoxic stress 168, illustrating a complex interplay between fission and mitophagy that still needs to be clarified.

Following mitochondrial fission and depolarization, Parkin and PINK1 (PTEN-induced putative kinase 1) are recruited to the OMM to tag the organelle for mitophagy. PINK1 is a serine/threonine kinase that identifies and targets specific mitochondria for degradation. Healthy mitochondria maintain a stable membrane potential that facilitates the import of PINK1 into the mitochondrial matrix, where it is cleaved and degraded 169. The proteolysis of PINK1 is mediated by the IMM-associated PARL protease, and regulated by the recently described SPY complex 170. However, upon impaired mitochondrial protein import, severely damaged mitochondria lack sufficient membrane potential to translocate PINK1, which is then stabilized in the OMM, recruiting Parkin from the cytosol. Parkin is an E3 ubiquitin ligase that, during mitophagy, poly-ubiquitinates multiple proteins to direct towards degradation. Parkin substrates include mitofusins Mfn1 and Mfn2, which are large GTPases that promote OMM fusion 171. Ubiquitination of Mfn1/2 prevents the refusion of the damaged mitochondria with the healthy mitochondrial network, and also signals for the recruitment of ubiquitin-binding proteins such as p62/SQSTM1, which mediate the aggregation into APs 172. p62/SQSTM1 acts as an ubiquitin-binding scaffold protein that undergoes disulfide bond-linked self-aggregation to interact with LC3 on autophagic membranes leading the co-delivery with its cargoes to the autophagosome 173. Nevertheless, mitophagy can occur independently of p62/SQSTM1, as recently reported by the Youle lab 174. Even though p62/SQSTM1 was required for Parkin-induced mitochondrial clustering, acute loss of p62 in HeLa cells by siRNA did not prevent Parkin-induced mitophagy, which indicates that p62/SQSTM1- independent mechanisms may mediate some events of the mitophagic process downstream of Parkin 174.

Mitochondrial ATP status and ΔΨ_m_ depolarization can also act as regulators of mitophagy by promoting Opa1 cleavage. Opa1 regulates mitochondrial fusion and cristae structure in the IMM 175,176. Mammalian cells express eight Opa1 splice forms 177, a proportion of which is constitutively cleaved by the YME1L protease, generating fusion-competent mixed populations of long and short OPA1 (L-OPA1 and S-OPA1, respectively) 178,179. Upon mitochondrial membrane depolarization, the long isoforms of Opa1 (L-OPA1) undergo cleavage by the protease OMA1, rendering the membrane incapable to sustain fusion. Interestingly, mitophagy was significantly impaired in OMA1 deficient cells, indicating that cleavage of L-OPA1 acts as a crucial control point for mitophagy 180.

**Figure 4:**
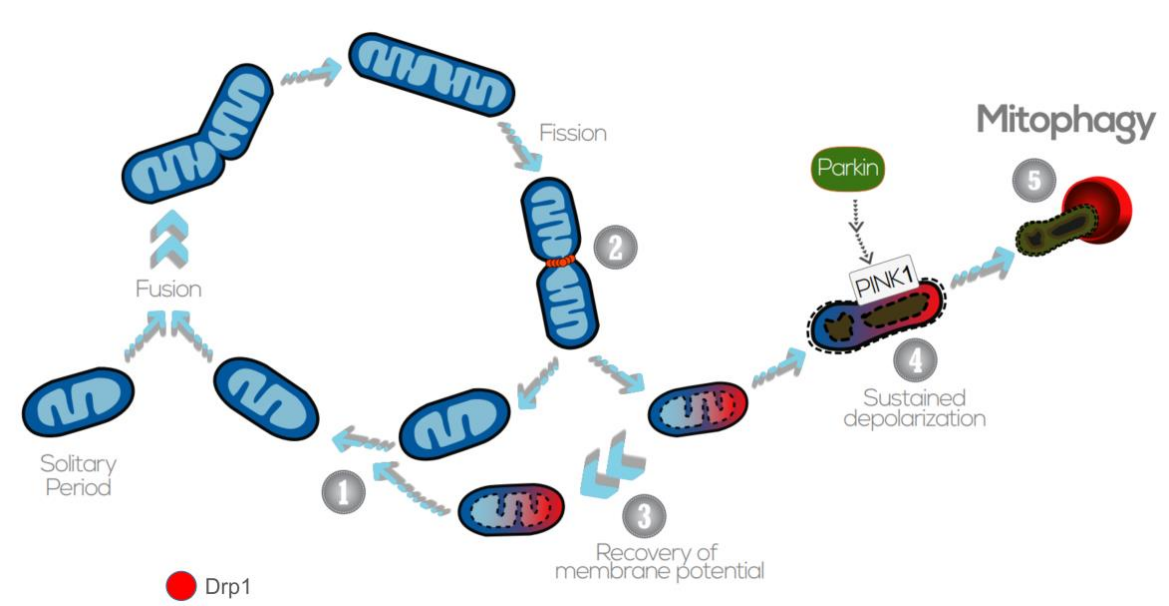
FIGURE 4: The interplay between mitochondrial dynamics and mitophagy. The mitochondrial network shifts between continuous cycles of fusion and fission, each of which can last seconds to minutes. Fission of mitochondria (2) is carried out by the Drp1 GTPase, which is recruited to the OMM from the cytosol. Fission events often generate daughter units with different ΔΨ_m_: on the one hand, some mitochondria are depolarized, but the ΔΨ_m _can be restored (3) by fusion with other mitochondria (1). After *solitary periods*, mitochondria might also fuse if the membrane potential is above a certain threshold. On the other hand, sustained ΔΨ_m_ depolarization triggers cleavage of Opa1, reduction in Mfn capacity and accumulation of PINK1/Parkin in the OMM (4). This targets single dysfunctional mitochondrion to mitophagy (5), where it is engulfed by the autophagosome. Image adapted from Twig G. and Shirihai O.S. (2011) 188.

Overall, sustained membrane depolarization results in cleavage of Opa1 in the IMM, accumulation of PINK1/Parkin and ubiquitination of Mfn1/2 in the OMM, and this promotes the damaged and targeted mitochondria to be engulfed by the AP (**Figure 4**). Nevertheless, it should also be noted that mitophagy can be modulated in a PINK1/Parkin - independent manner, upon hypoxia or targeting by other OMM proteins. For instance, Nix is an OMM receptor that activates the apoptotic machinery and favors the elimination of mitochondria during red blood cell differentiation and elimination in erythrocytes 181. Nix contains a LIR (LC3-interacting region) and, upon activation, Nix and BNIP3 promote the opening of the mitochondrial transition pore, which results in depolarization of the organelle and recruitment of LC3/GABARAPs for autophagosome formation 182,183. In addition to Nix, FUNDC1 - an integral OMM protein - modulates mitophagy in response to hypoxia by the increased affinity between its LIR motif and LC3 on AP membranes that results after FUNDC1 phosphorylation by ULK1 184. FUNDC1-mediated mitophagy has been recently demonstrated to play an essential role in cardiac function *in vivo* 185. Iron chelators can also generate a mitophagy response in the absence of PINK1, yet the mechanism requires to be further elucidated 186. Of note, basal mammalian mitophagy occurs independently of PINK1 in tissues with high metabolic demand, which could indicate that mammalian cells have multiple mitophagy pathways that can be triggered in response to diverse stress stimuli 187.

Interestingly, the UPR^mt ^and the mitophagic events could be connected through a common stressor mechanism which might benefit the mitochondrial population that can still be recovered over the one that is targeted for mitophagy. While mitophagy limits the damage to defective mitochondria, stress responses such as the UPR^mt ^facilitate the recovery of salvageable mitochondria, ultimately yielding a healthier mitochondrial network 91. As Pickles (2018) suggested, the mitochondria that sends ATFS-1 to the nucleus may not benefit from the action of the chaperones and proteases that could be imported into more fit mitochondria, leaving the most damaged mitochondria for mitophagy 189.

Although dysfunctional mitochondria that are fragmented are more likely to be targeted for mitophagy, some species-dependent exceptions can be found 161,190. On the other hand, daughters of mitochondrial fission have unequal probabilities of undergoing subsequent fusion. Fusion preferentially occurs between mitochondria with higher ΔΨ_m_, as they are presumed to have better quality that can help maintain the activity of the network. This establishes mitophagy and fusion as two competing events determining the fate of the mitochondria.

## MITOCHONDRIAL FUSION AS A SELECTIVE RESCUE MECHANISM

Mitochondrial fusion allows the diffusion of matrix and membrane components. This serves as a ‘compensatory’ mechanism to equilibrate proteins, complexes and metabolites from one healthy mitochondria to a second one that might be damaged. Hence, fusion may recruit dysfunctional mitochondria into the active pool, instead of being eliminated by mitophagy.

Mitochondrial fusion contributes to the maintenance of a stable ΔΨ_m, _homogeneity of the components of the electron transport chain and stability of mtDNA nucleoids. Heteroplasmic cells - with both normal and mutant mtDNA - can accumulate mutations to a threshold level before respiratory activity is affected and the mitochondria is targeted for mitophagy. Reaching this turnover would be relented by the compensatory mechanisms that mitochondrial fusion brings by complementing mtDNA from healthy organelles. Real time imaging experiments by Yang *et al*. (2015) allowed the visualization of mtDNA nucleoid dynamics after complete fusion of the mitochondria. Rho0 cells recovered their ΔΨ_m_ after fusion with wild type cells, and this was prevented by the deletion of Mfn1/2 and Opa1. Interestingly, motility of the newly fused mitochondria was increased 191, which would allow specific positioning of the mitochondria for different metabolic and cellular processes. Fusion compensatory function was also validated in skeletal muscle of Mfn1/2-deficient mice 192,193. The rate of mtDNA point mutations and deletions was increased in these mice, events preceded by physiological abnormalities and muscle atrophy. Moreover, stimulation of mitochondrial motility by overexpression of Miro-1 - a mitochondrial Rho-GTPase that promotes mitochondrial movement along microtubules - increases mitochondrial fusion in neurons 194. Mitochondria proximity favored by Ca^+2^ oscillations also contributes to increased fusion rate 195: the closer two mitochondria are, the better chances for them to fuse. Fusion events are also dependent on the intermediates of the metabolic processes taking place in the mitochondrial matrix, as observed by Cavellini *et al*. (2017), reporting that unsaturated fatty acids impede outer membrane fusion, establishing a mechanistic crosstalk between mitofusins and fatty acid desaturation 196. Similarly, high OXPHOS levels can stimulate mitochondrial inner membrane fusion by increased efficiency of YME1L in the proteolytic processing of Opa1 197.

Then, what determines whether mitochondria should be rescued by fusion from autophagy? Redistribution has an impact upon mitochondria function. Non-selective fusion would contribute to damaged mitochondria impairing the activity and efficiency of the healthy population. It was demonstrated by Twig *et al*. (2008) that, after fission, fusion occurs preferentially between mitochondria with higher ΔΨ_m_, as compared to the subpopulation of non-fusing mitochondria that presented depolarized ΔΨ_m_. The depolarization of an individual mitochondrion is also accompanied by reduced levels of Opa1, and this decreases its probability of undergoing fusion 163. These findings would indicate that fusion is a selective and exclusive process for hyperpolarized mitochondria, rather than an unselective rescue mechanism. Thus, these two traits, reduced ΔΨ_m _and loss of Opa1 activity, would promote targeting of mitochondria for autophagy. The selectivity of mitochondrial fusion not only prevents the migration of damaged components into active mitochondria, but is also an isolation step that creates a segregated population that is available for autophagy (**Figure 4**).

Nevertheless, fusion is not only a compensatory, but also a protective mechanism of mitochondria against metabolic and stress challenges, carried out by modification of the key players in the fusion machinery. Upon starvation, the increase in cAMP levels activates PKA, which phosphorylates Drp1 at Ser637 and keeps it in the cytosol, leading to unopposed mitochondrial fusion. Elongated mitochondria are spared from autophagic degradation, present higher oligomerization of ATPase and increased efficiency of ATP production. Therefore, mitochondrial elongation during starvation protects cells from death 144. Furthermore, mitochondrial fusion after fasting eliminates oxidative stress via association of Mfn1 with the protein deacetylase HDAC6, which leads to Mfn1 deacetylation and activation 144. Accordingly, HDAC6 knockout mice showed impaired mitochondrial fusion capacity upon glucose deprivation, resulting in mitochondrial degeneration, excessive production of ROS and oxidative damage in muscle 144.

Mitochondrial fusion has been suggested to provide a new treatment for mitochondria-related diseases such as diabetes, muscular dystrophies or neurodegenerative disorders, hence the increasing interest in developing activators of mitochondrial fusion 198. In this regard, leflunomide - a drug approved to treat rheumatoid arthritis - has recently been documented to increase Mfn2 expression and mitochondrial fusion 199. A recent report indicates that the conformation of Mfn2 heptad repeat domains is critical for Mfn2 GTPase activity. A close conformation is fusion incompetent, whereas an open conformation favors mitochondrial fusion. This open conformation can be induced by a competing peptide analogous to amino acids 367 to 384 within the Mfn2, resulting in the activation of Mfn2 and enhanced mitochondrial fusion. This new class of mitofusin agonists have been shown to ameliorate mitochondrial motility and depolarization in neurological disease models 200. These works bring hope on the therapeutic possibilities of enhancing mitochondrial fusion in models characterized by fragmented, depolarized mitochondria.

Therefore, the elimination of damaged mitochondria by mitophagy and the elongation of the functional mitochondrial network by fusion could act as complementary processes to guarantee cell homeostasis and disease prevention. The choice of one or another to preserve mitochondrial health might depend on multiple parameters, including the degree of mitochondrial damage, the basal mitochondrial turnover rate or the intrinsic ability of the tissue/cell type to mobilize mitochondria for degradation.

## MITOCHONDRIAL BIOGENESIS

Mitochondrial biogenesis is the process of increasing cellular mitochondrial mass. In most cases, mitochondrial biogenesis occurs in response to energy deficit, triggered either by increased cellular energy demand or by impaired ATP synthesis. Hence, it is not surprising that both situations share, at least in part, common mechanisms to increase mitochondrial copy number.

One possible starting point for mitochondrial biogenesis in situations of energy stress might be found in the activation of the AMP-activated protein kinase (AMPK). AMPK is a heterotrimeric enzyme composed by an α, β and γ subunit, all of which can be present as different isoforms 201. The γ subunit acts as a sensor for the AMP/ATP ratio 201, providing AMPK an extremely refined capacity to respond to alterations in the cellular energy status. Upon activation, AMPK - or any of its eukaryote homologs - acts as a master metabolic controller in the cell 202. In particular, AMPK shuts down most energy consuming programs not necessary for the immediate survival of the cell, including cellular division, cellular growth and most anabolic paths. On the other side, AMPK activates multiple cellular processes aimed to enhance energy production, such as increasing glucose uptake and glycolytic and fatty acid oxidation fluxes. Interestingly, AMPK activation can also trigger long-term adaptations, being mitochondrial biogenesis one of its defining features 203.

AMPK influences mitochondrial biogenesis, in part but not exclusively, through the activation of the transcriptional coactivator PGC-1α (peroxisome proliferator activator receptor gamma coactivator 1a). PGC-1α was originally described as a cold-induced transcriptional coactivator in brown adipose tissue 204, but has been later certified as a master regulator of mitochondrial biogenesis 205. This way, AMPK activation enhances mitochondrial and lipid oxidation markers in most cells tested to date. AMPK activators, however, failed to enhance mitochondrial-related gene expression and protein markers content in PGC-1α deficient models 206,207. Several mechanisms by which AMPK influences PGC-1α have been proposed. First, AMPK activation can lead to a transcriptional increase in PGC-1α levels 208,209. Second, it was described that AMPK can directly phosphorylate and activate PGC-1α coactivation properties 207. It has also been proposed that the phosphorylation of PGC-1α by AMPK might not trigger activation *per se*, but might alter the ability of PGC-1α to interact with other proteins 210. Finally, there is evidence suggesting that the direct phosphorylation of nuclear receptors and transcription factors by AMPK could influence the recruitment of PGC-1α 211. Most of these actions are not necessarily contradictory and could take place simultaneously.

Interestingly, AMPK influences the activity of another key regulator of cellular metabolism and mitochondrial function, the NAD^+^-dependent protein deacylase SIRT1 210,212. The Km of SIRT1 for NAD^+^ is high enough to make physiological intracellular levels of NAD^+^ rate-limiting for SIRT1 activity 213. This transforms SIRT1 into a potential NAD^+^ sensor in the cell, hence responding to metabolic and oxidative stress challenges. AMPK has been shown to increase intracellular NAD^+^ levels through metabolic rewiring upon fatty acid availability 210 and through transcriptional means 212, leading to SIRT1 activation. SIRT1, in turn, can deacetylate and activate PGC-1α as well, leading to its activation 214. In fact, AMPK-induced PGC-1α phosphorylation has been proposed to serve as a way to facilitate PGC-1α recognition by SIRT1 210. SIRT1 can also deacetylate other transcription factors and nuclear receptors controlling mitochondrial and fatty acid-related gene expression, O_2_ delivery or oxidative stress defenses, including PPARα, ERRγ and Forkhead-O-box transcription factors 213.

PGC-1α, however, is a nuclear coactivator, and mitochondrial biogenesis requires the replication and transcription of the mitochondrial genome. Bringing light into these issues, it was shown that the nuclear expression of the mitochondrial transcription factor A (TFAM), the key controller of mitochondrial DNA replication, is controlled via the nuclear respiratory factors 1 and 2 (NRF-1 and NRF-2) 215. In fact, NRF-1 and NRF-2 bind to most of the promoters encoding for subunits of the mitochondrial respiratory chain 215.

AMPK exemplifies how a mitochondrial-related cue (energy balance) can be transformed into a metabolic adaptation. Nevertheless, mitochondrial biogenesis and PGC-1α can be controlled through other means, for examples in situations of cancer or cellular proliferation 216. Similarly, mitochondrial biogenesis cohabitates with other responses. For example, SIRT1 activation has been shown to trigger the UPR^mt^ 217 which makes sense, as mitochondrial repair and biogenesis could constitute coordinated activities aimed to ensure mitochondrial fitness. Finally, mitochondrial biogenesis is not solely controlled by PGC-1α. Indeed, PGC-1α deficiency in mice does not lead to major impairments in baseline mitochondrial function, and multiple eukaryote species and organisms, including yeast or worms, do not have PGC-1α homologs, yet they can trigger mitochondrial biogenesis in response to nutrient availability. Nevertheless, while multiple paths might control the expression of mitochondrial-related genes, PGC-1α might have appeared as a key modulator to achieve flexible solutions.

## CONCLUDING REMARKS

Mitochondria are a key source for fuel and intermediate metabolites that are critical for cellular proliferation, differentiation, growth and function. The clear link between mitophagy and human diseases suggests a potential applicability of this process as a therapeutic target. However, some aspects need to be taken into consideration for the development of therapeutic strategies, including the tissue to be targeted or the severity of the disease. In physiological conditions, mitochondrial turnover takes place at very different rates depending on the tissue examined, and may even vary in the mitochondrial population of tissues of the same type 218. This suggests that the key players in mitophagy and mitochondrial dynamics regulation could be dependent on the tissue and stimuli they are exposed to.

The different mechanisms ensuring mitochondrial quality control may gradually enter into scene, starting by basic defense systems such as the antioxidant responses against oxidative damage resulting from increased production of ROS. When these defenses are not sufficient and affect protein function and folding, mitochondria communicates to the nucleus to activate the unfolded protein response mediated by chaperones and proteases to restore protein homeostasis. Mitochondrial dynamics and mitophagy will also act reciprocally to maintain the quality, not just of individual organelles, but of the entire mitochondrial network. When this process is compromised, different pathologies arise, out of the accumulation of dysfunctional mitochondria or defective fusion/fission mediators. It must be clarified, however, that many of these mechanisms might occur simultaneously in a mitochondrial population, or even in the same mitochondrion. The boundaries and independence of these responses are therefore unclear. Similarly, some mitochondrial stress responses, such as the mammalian UPR^mt^ are still too vaguely defined. It will be important to define a clear standardization in the evaluation of UPR^mt ^as, in fact, most works do not characterize mitochondrial protein aggregation.

Studying organelles in isolation has been indispensable for constructing our main body of knowledge on mitochondrial physiology. However, mitochondria do not behave as isolated entities in the cytosol, but as signaling organelles that communicate continuously with other cellular membranes and within themselves. Mitochondria impact nuclear gene expression by retrograde signaling, but also influence metabolite transfer through the physical contact sites with other organelles, such as the ER, lipid droplets, peroxisomes and lysosomes 219. Decoding the intricate network of relations and responses elicited by mitochondria does not only constitute a riveting scientific challenge, but also the opening of new potential understanding for therapeutic approaches. Further, the very different behavior and characteristics of mitochondria in each tissue constitute a promising way to approach tissue-specificity in potential mitochondrial treatments. Recent findings even demonstrate that within a single cell there might be specific mitochondrial populations with dedicated bioenergetic capacities 220. Hence, to understand the therapeutic potential around mitochondria, we will need to understand their identity and function, but also the time and space in which they participate within the cell life.

## Abbreviations

α-KG: α-ketoglutarate
Aβ: amyloid β peptide
AMPK: AMP-activated protein kinase
AP: autophagosome
CAT: catalase
CHOP: C/EBP homologous protein
CLPP: ATP-dependent caseinolytic protease proteolytic subunit
COX: cytochrome C oxidase
ER: endoplasmic reticulum
GPx: glutathione peroxidases
IMM: inner mitochondrial membrane
IMS: intermembrane space
ISR: integrated stress response
MDP: mitochondrial derived peptide
MSR: mitochondrial stress response
mtDNA: mitochondria DNA
OMM: outer mitochondrial membrane
OXPHOS: oxidative phosphorylation
PERK: protein kinase RNA-like ER kinase
PGC-1α: Peroxisome Proliferator Activator Receptor Gamma Coactivator 1α
PINK1: PTEN-induced putative kinase 1
ROS: reactive oxygen species
SOD: superoxide dismutase
TCA: tricarboxylic acid cycle
TrRx: Thioredoxin reductases
UPR: unfolded protein response
ΔΨ_m_: mitochondrial membrane potential
